# Role of Bone Marrow Examination in the Evaluation of Infections: Clinico-Hematological Analysis in a Tertiary Care Centre

**DOI:** 10.5146/tjpath.2019.01466

**Published:** 2020-01-15

**Authors:** Vijay Kumar, Akanksha Bhatia, Garima Baweja Madaan, Sadhna Marwah, As Nigam

**Affiliations:** Department of Pathology, PGIMER, Dr. Ram Manohar Lohia Hospital, New Delhi, India

**Keywords:** Bone marrow examination, Infections, Pyrexia, Tuberculosis, Visceral leishmaniasis, HIV

## Abstract

***Objective:*** Bone marrow examination (BME) is an important modality for investigation of case of pyrexia of unknown origin (PUO). However, its yield in the diagnosis of infections has not been extensively studied and its role has not been well established. The aim of the study was to investigate the usefulness of BME and to evaluate the etiological and clinico-hematological profile in cases of bone marrow infections.

***Material and Method:*** This was a retrospective study where bone marrow cases were retrieved and a review of bone marrow findings with an infectious etiology from July 2014 to June 2018 was done. Detailed history, clinical examination and hematological parameters at presentation were recorded. Clinico-hematological correlation using descriptive statistics was performed.

***Results:*** The study included 55 cases, on analysis of which the maximum number of infections were those of leishmaniasis accounting for 35%, followed by HIV (29%) and tuberculosis (15%). Other etiological agents included fungal infections (histoplasmosis and aspergillosis), Enteric fever, Scrub typhus, parvovirus, falciparum malaria and filariasis. The most common clinical presentation was fever (80%) and the most common clinical finding was splenomegaly (66%).

***Conclusion:*** Bone marrow examination is an important diagnostic tool to delineate etiological diagnosis in infectious conditions, particularly those presenting with PUO. Moreover, it is particularly important if urgent diagnosis is required or if alternate diagnostic modalities have not revealed a reason for PUO.

## INTRODUCTION

Bone marrow examination (BME) plays an important role in diagnosis of hematological as well as non-hematological disorders. It is a simple and safe procedure and is particularly useful in the investigation of pyrexia of unknown origin as it leads to an etiological diagnosis in most of the cases (1,2). Anemia and other peripheral cytopenias are the most frequent indications for bone marrow examination and may be the presenting signs of a clinically unsuspected infection identifiable within the bone marrow.

Bone marrow changes resulting from infections can be studied by analysis of morphology and etiology. On morphology, similar lesions can be seen to arise from different infectious agents and one agent can give rise to various lesions (3). Routine staining procedures (Giemsa) may be very helpful in the diagnosis of viral inclusions, some parasites (*Leishmania*, *Toxoplasma*, *Microfilaria*) and fungi (*Histoplasma*, *Cryptococcus*). Sometimes special stains like Ziehl-Neelsen (ZN) and periodic acid-Schiff (PAS) stains may be required to identify organisms like *Mycobacterium* and certain fungi.

The yield of bone marrow examination in the diagnosis of infections has not been extensively studied and its usefulness has not been well established. Hence, a retrospective study was performed to investigate the role of BME along with clinico-hematological analysis; and to help clarify its role in the diagnosis of various infections.

## MATERIALS and METHODS

The study was a retrospective study conducted in the Department of Pathology. All case records of bone marrow examination were retrieved and the bone marrow findings were reviewed from July 2014-June 2018; and those diagnosed as infections were included in the study. Clinical details, biochemical profile, complete hemogram with peripheral smear, bone marrow aspiration smears and bone marrow biopsy slides (wherever available) were reviewed and data were analyzed. All cases where bone marrow aspiration/biopsy was inadequate for an opinion were excluded from the study. Institutional ethical clearance was not obtained as it was a retrospective study.

Peripheral blood smear was routinely examined along with the bone marrow aspiration and biopsy slides and cytopenia was defined as: hemoglobin < 10 gm/dL, total leucocyte count <4×103/µL and platelet count<100×103/µL. Bone marrow aspiration and trephine biopsy had been carried out as per the clinical indication. The bone marrow procedure was carried out by standard methods. All the bone marrow aspirate smears and trephine biopsies were stained with Giemsa and hematoxylin and eosin (H&E). In addition, wherever indicated, PAS and ZN stains were applied to aspirate smears and biopsies. Bone marrow culture was not available in these cases. Statistical analysis using descriptive statistics was done.

## RESULTS

The study included 58 cases, out of which 3 samples were hemodiluted and hence were excluded from the study. Of the remaining 55 cases, bone marrow aspiration and biopsy had been performed for 27 cases (49%); whereas only aspiration had been done in 28 cases (51%). There were 42 (76%) cases of adults and 13 (24%) of children; and the mean age of presentation was 32.3 years (range, 1-72 years). The male:female ratio was 2.2:1. The clinical presentation of the cases varied from high grade fever and abdominal pain to non-specific symptoms like generalized weakness (Table 1). Fever was the commonest symptom and seen in 80% of the cases (n=44).

**Table 1:** Clinical presentation of the cases included in the study.

**Clinical presentation**	**Number of cases (%)**
Fever	44 (80)
Abdominal pain	12 (22)
Vomiting/diarrhea	6 (11)
Bleeding manifestations	6 (11)
Generalised weakness/arthralgia	7 (13)
Cough/Breathlessness	9 (16)
Weight loss	3 (6)

On clinical examination, splenomegaly was the commonest finding, seen in 66% of the cases (n=36). Other significant findings were hepatomegaly, lymphadenopathy, effusion (ascitic/pleural/pericardial) and pallor (Table 2). The HIV antigen was found to be positive in 29% of the cases (n=16) and the RK39 antigen for leishmaniasis was positive in 16% of the cases (n=9).

**Table 2:** Clinical examination and investigations of the cases included in the study.

**Clinical findings**	**Number of cases (%)**
Splenomegaly	36 (66)
Hepatomegaly	28 (51)
Lymphadenopathy	7 (13)
Effusion	7 (13)
Pallor	6 (11)
HIV antigen (+)	16 (29)
RK39 (+)	9 (16)
Rickettsial antigen	2 (4)
Parvo IgM (+)	2 (4)
Widal test (+)	2 (4)
Typhidot (+)	1 (2)

On peripheral smear examination, anemia was the most common finding seen in 91% of the cases (n=50). The most common type of anemia was the normocytic normochromic type. Other findings were pancytopenia, leucopenia, thrombocytopenia and rouleaux formation. Common hematological findings are summarized in Table 3.

**Table 3:** Common hematological findings observed in the study.

	**Number of cases (%)**
Pancytopenia	17 (31)
Anemia	50 (91)
Leucopenia	32 (58)
Thrombocytopenia	20 (36)
Rouleaux formation	2 (4)
Neutrophilia	6 (11)
Eosinophilia	2 (4)
Lymphocytosis	1 (2)

**Figure 1:**
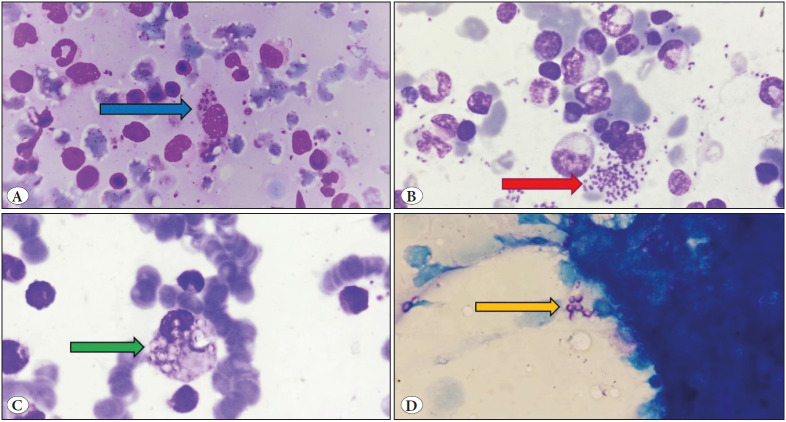
**A,B)** Visceral leishmaniasis: Bone marrow aspirate showing presence of intracellular (A, blue arrow) and extracellular (B, red arrow) LD bodies (Giemsa; x1000). **C,D)** Histoplasmosis: Bone marrow aspirate showing presence of intracellular (C, green arrow; Giemsa; x1000) and extracellular (D, yellow arrow; PAS; x1000) histoplasma.

**Figure 2:**
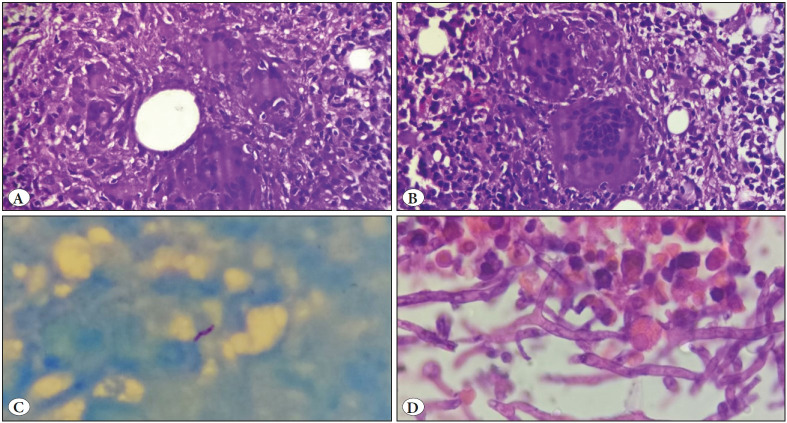
**A-C)** Tuberculosis: Epithelioid cell granulomas on bone marrow biopsy seen in a case of HIV (A,B; H&E; x400) and acid fast bacilli seen on ZN stain (C; ZN; x1000). **D)** Aspergillosis: Bone marrow biopsy showing presence of numerous acute angle branching hyphae of Aspergillus (H&E; x1000).

**Figure 3:**
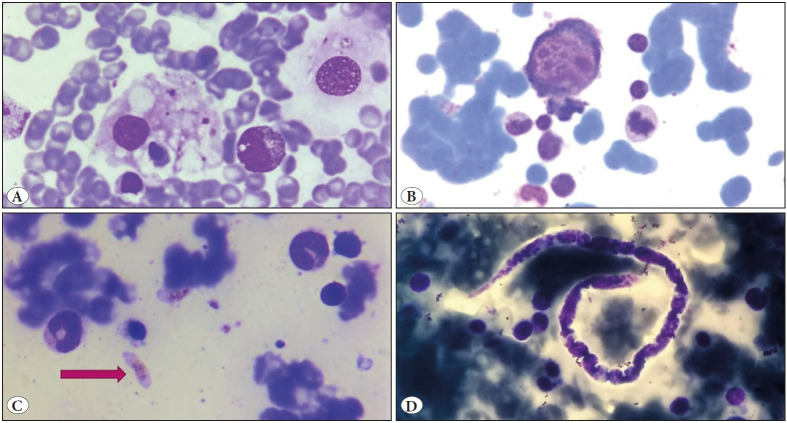
**A)** Bone marrow aspirate showing presence of hemophagocytosis in a case of Scrub typhus (Giemsa; x1000). **B)** Giant proerythroblast with nuclear inclusion and erythroblastopenia seen in a case of Parvovirus infection (Giemsa; x1000). **C)** Gametocytes of Plasmodium falciparum seen on bone marrow aspirate seen in a case of Malaria (arrow; Giemsa; x1000). **D)** Bone marrow aspirate showing Microfilaria (Giemsa; x200).

**Figure 4:**
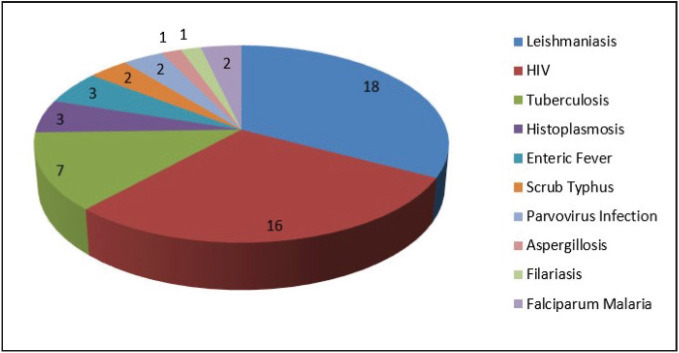
Spectrum of infections observed in the study.

On bone marrow examination, an increase in plasma cells and histiocytes along with hemophagocytosis was commonly observed. Erythroid hyperplasia and dyserythropoiesis were also commonly seen (Table 4). A total of 19 cases showed presence of *Leishmania donovani* (LD) bodies (Figure 1). Granulomas were seen in 8 cases; however acid fast bacilli could be demonstrated only in 1 case on ZN staining (Figure 2). PAS positive histoplasma was observed in 3 cases (Figure 1). Giant proerythroblasts having intranuclear inclusions (Figure 3) suggestive of parvo virus infection; and gametocytes of Plasmodium falciparum (Figure 3) were seen in two cases each. There were one case from each of aspergillosis (Figure 2) and microfilariasis (Figure 3). Erythroblastopenia and erythrophagocytosis were also observed. The spectrum of infections observed in the study is demonstrated in Figure 4.

**Table 4:** Common bone marrow findings in the study.

	**Number of cases (%)**
Erythroid hyperplasia	32 (58)
Dyshemopoiesis	13 (24)
Erythroblastopenia	2 (4)
Eryhtrophagocytosis	3 (5)
Plasmacytosis	25 (46)
Increase in histiocytes	5 (9)
Increase in eosinophils	5 (9)
Lymphocytosis	5 (9)
Hemophagocytosis	10 (18)
LD bodies	19 (35)
Granuloma	8 (15)
Histoplasma	3 (5)
Malarial parasite	2 (4)
Aspergillus hyphae	1 (2)
Microfilaria	1 (2)

## DISCUSSION

A variety of morphologic changes in the bone marrow have been described in various infectious. These changes may be features of acute inflammation (interstitial edema, vascular congestion, hemorrhage, ischemic necrosis or suppurative necrosis) or chronic inflammation with granuloma formation, reactive lymphoid hyperplasia, plasmacytosis, histiocytosis or fibrosis (3). Bone marrow examination can also lend to the etiological diagnosis in many cases.

Many studies have demonstrated the utility of bone marrow in a particular type of infection; however no study has been done which has studied the spectrum of infections which can be seen on bone marrow examination (4,5).

In this study, there were 19 cases (35%) where amastigote forms of LD bodies were seen. Of these, there was one case of HIV infection which developed visceral leishmaniasis, and showed presence of granulomas with LD bodies even on FNAC of cervical lymph node. In the bone marrow aspirates, LD bodies were mostly seen both intracellularly and extracellularly (89.5%). Corresponding bone marrow biopsy was available in 8 of these cases, of which LD bodies were seen in 6 cases (75%). Other findings associated with leishmaniasis were reactive plasmacytosis in the bone marrow seen in 79% of the cases and rouleaux formation on peripheral smear seen in 2 cases. Clinically, fever was the most common presenting symptom (95%) and splenomegaly was seen in all cases. This is in accordance with various studies and case reports of visceral leishmaniasis (6). In a study conducted by Chandra et al. similar findings have been observed. In their study, fever was the most common presenting feature followed by hepatosplenomegaly (7). Plasmacytosis and hemophagocytosis were common findings and were attributed to the longer duration of the symptoms (8).

There were 16 cases (29%) of diagnosed HIV infection for which bone marrow examination was done. Out of these, opportunistic infections like tuberculosis was seen in one case (granulomas with positive ZN stain) and presence of LD bodies in another. Two cases showed presence of malignancy (T-cell lymphoma and acute leukemia). One case showed therapy related changes in the bone marrow and one case was hypocellular with focal gelatinous changes. The rest of the HIV positive cases showed reactive changes like reactive plasmacytosis and prominence of histiocytes. Dyshemopoiesis in the form of dyserythropoiesis was seen in most of the cases. However, the diagnostic yield of bone marrow in HIV infections in the present study was only 31%. This is similar to study conducted by Pande et al. where the diagnostic yield of bone marrow was 26% (9). However, in the present study there were only 2 cases (13%) of superadded infections being diagnosed on bone marrow and fungal infections were not identified. This is in contrast to the above-mentioned study, where superadded infections accounted for 50% of the cases. However, there were two cases of malignancy (T-cell lymphoma and acute leukemia) which also accounted for 13% of the total HIV cases. Other non-specific findings like dysplastic changes and plasmacytosis have been reported in various other studies as well (9,10).

There were 8 cases (15%) of tuberculosis in our study, including the one seen in a HIV positive patient. Of these 8 cases, 5 of them showed granulomas only in bone marrow biopsy; one case showed granulomas both in aspirate and biopsy; and 2 cases showed granuloma only in the marrow aspirate. However, ZN stain was positive only in one case. This may be due to the fact that acid fast bacilli are seen only in 25% of marrow biopsies as claimed by other studies (11). So, in a country like India it is difficult to rule out tuberculosis in the absence of any associated findings with the granuloma. Cases with caseous necrosis were also seen in 3 cases (38%). This is similar to findings observed by Gupta et al. (6).

Fungal infections accounted for 4 cases (7%) of which 3 were that of histoplasmosis. Of these 3 cases, 2 presented with fever and one with pancytopenia. PAS positive *Histoplasma* organisms were seen mainly intracellularly in two cases and both intra- and extracellularly in one case. In a study conducted by Chandra et al., similar findings were observed in a case of histoplasmosis on bone marrow aspirate; however they have also described hemophagocytosis as a clue towards diagnosing the disease (12). In the present study, however, hemophagocytosis was not observed in any of the cases. There was one case of aspergillosis where PAS positive acute angled fungal hyphae were seen in the bone marrow biopsy. Serology for HIV was negative in all of the four cases. This is unlike other studies wherein fungal infections in the marrow have been mostly described in HIV positive cases (13).

Other than these above mentioned infections, there were 3 cases of enteric fever where erythrophagocytosis was evident in the marrow. However, no granulomas were seen. Two of these cases turned out to be WIDAL positive and one case was Typhidot positive. This is in accordance with many studies where presence of erythrophagocytosis without granuloma was seen in cases of enteric fever (14). There were 2 cases of scrub typhus which showed evidence of hemophagocytosis with mild dyserythropoiesis.

Two cases of parvo virus infection (parvo IgM positive) were also included in the study, and showed paucity of erythroid precursors with giant proerythroblasts having intranuclear inclusions on bone marrow aspirate. These findings were similar to those described in earlier studies (15). However, B19 DNA PCR was not done in the present case. Two cases of falciparum malaria were also observed; wherein gametocytes of *Plasmodium falciparum* and intracellular hemozoin pigment within the histiocytes were observed. There was also a case of filariasis that presented with fever and where microfilaria were identified on bone marrow aspiration.

As the bone marrow aspiration and biopsy was performed by the clinical departments, bone marrow biopsy had not been done in 28 of the cases. This could partly be due to technical reasons. Nevertheless, bone marrow aspirates were adequate in those cases and a diagnosis could be established.

In conclusion, bone marrow aspiration and biopsy can be useful in diagnosing a variety of infections including bacterial, fungal, parasitic and even viral, in conjunction with the clinical findings. This study assumes more significance particularly in those cases where bone marrow culture is not available or remains non-contributory.

Bone marrow examination is an important diagnostic tool to delineate etiological diagnosis in infectious conditions, particularly those presenting with PUO. Moreover, it is particularly important if urgent diagnosis is required or if alternate diagnostic modalities have not revealed a reason for PUO. This study highlights the role of bone marrow examination as an important diagnostic modality for the etiological diagnosis of infection and thereby, helps to provide better management of such cases. To the best of our knowledge, no similar comprehensive study elucidating the role of bone marrow examination in the diagnosis of infections has been found in the literature.

## Conflict of Interest

The authors declare no conflict of interest.

## FUNDING

The authors received no specific funding for this work.
